# Supplementary figures and images for: Inferring a complete genotype-phenotype map from a small number of measured phenotypes
Source: PLoS Comput Biol. 2020 Sep 29;16(9):e1008243. doi: 10.1371/journal.pcbi.1008243 (PMC7546491; doi:10.1371/journal.pcbi.1008243)

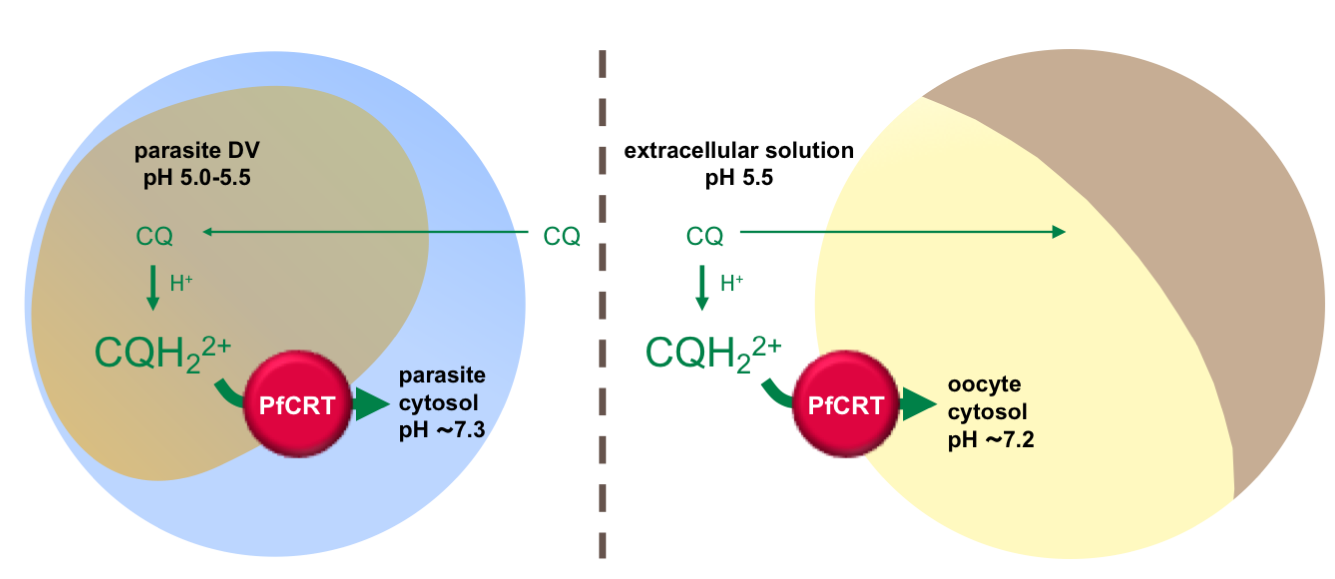

Supplement: S1 Fig — The orientation of PfCRT in both the parasite’s digestive vacuole (DV) membrane and in the oocyte plasma membrane is such that its N- and C-termini are located in the cytosol. Chloroquine (CQ) is a weak-base drug which, in its neutral form, can diffuse across the membranes of the parasitized erythrocyte and into the DV. Within the acidic environment of this compartment, CQ becomes protonated (CQH+ and CQH22+) and thereby accumulates via weak-base trapping. Protonated CQ is then effluxed from the DV, via mutant variants of PfCRT, into the parasite’s cytosol. In the Xenopus oocyte system, tritiated CQ is added to the acidic extracellular solution and the protonated drug is then transported into the oocyte’s cytosol via mutant variants of PfCRT. Diffusion of uncharged CQ into the oocyte also occurs, albeit to low levels. Note that in both scenarios, the direction of PfCRT-mediated CQ transport is from the DV lumen/extracellular solution into the cell cytosol. That is, CQ is translocated to the cytosolic compartment of the cell, which is also where the N- and C-termini of the transporter are located. (TIF) [file pcbi.1008243.s001.tif]

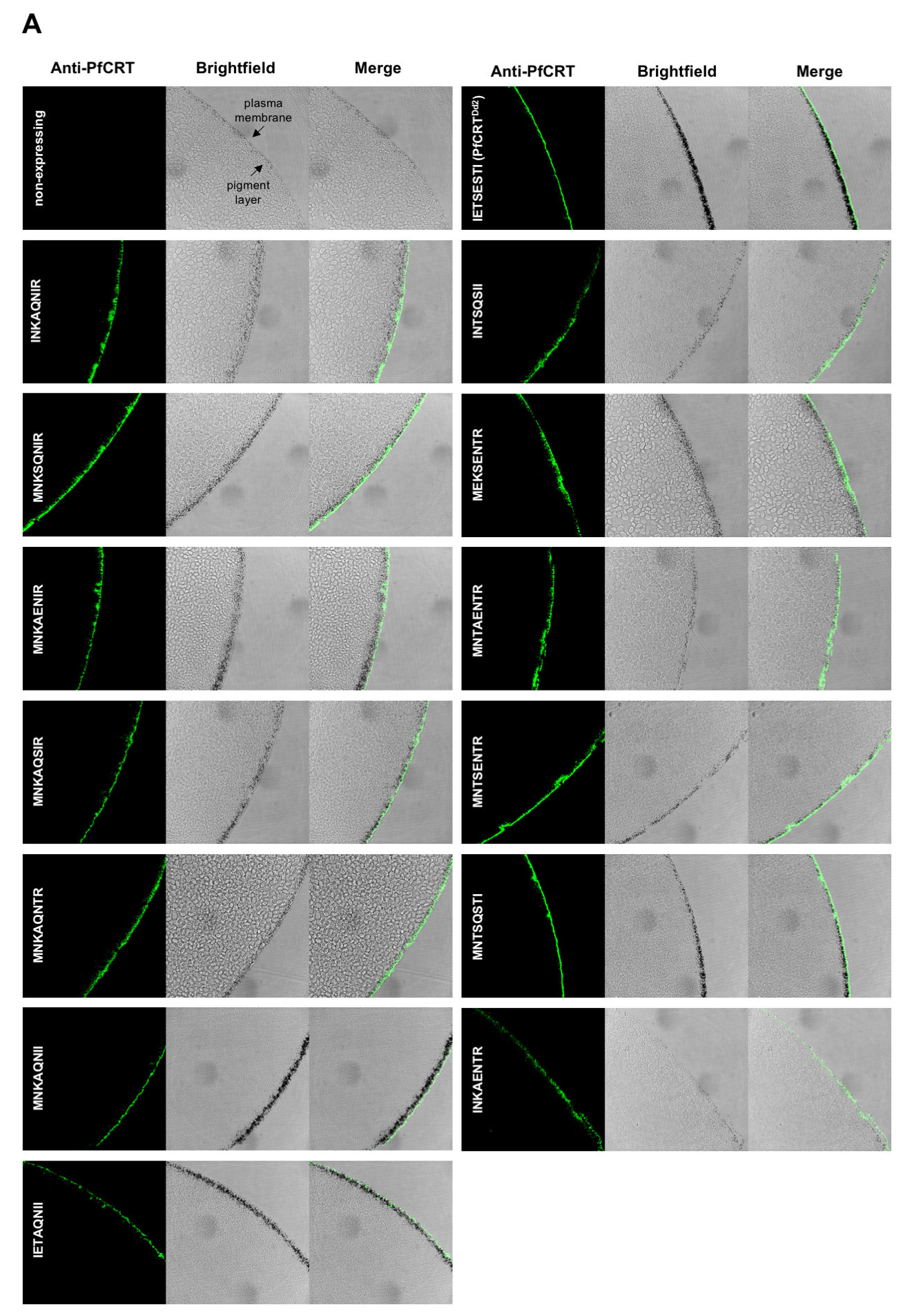

Supplement: S2 Fig — (Similar results for a second frog a shown in S3 Fig). Immunofluorescence microscopy was used to localize those PfCRT variants that exhibited little or no CQ transport activity when expressed in the oocyte system. In each case, the expression of the PfCRT variant resulted in a fluorescent band external to the pigment layer, indicating that the protein was expressed in the oocyte plasma membrane. The band was not present in non-expressing oocytes. Panels A and B show the images from two independent experiments that were performed using oocytes from two different frogs, and within which images were obtained from a minimum of three oocytes per PfCRT variant. Refer to Richards et al. [75] for the localization of IEKSESII (i.e. the ‘106/1’ isoform of PfCRT). (TIF) [file pcbi.1008243.s002.tif]

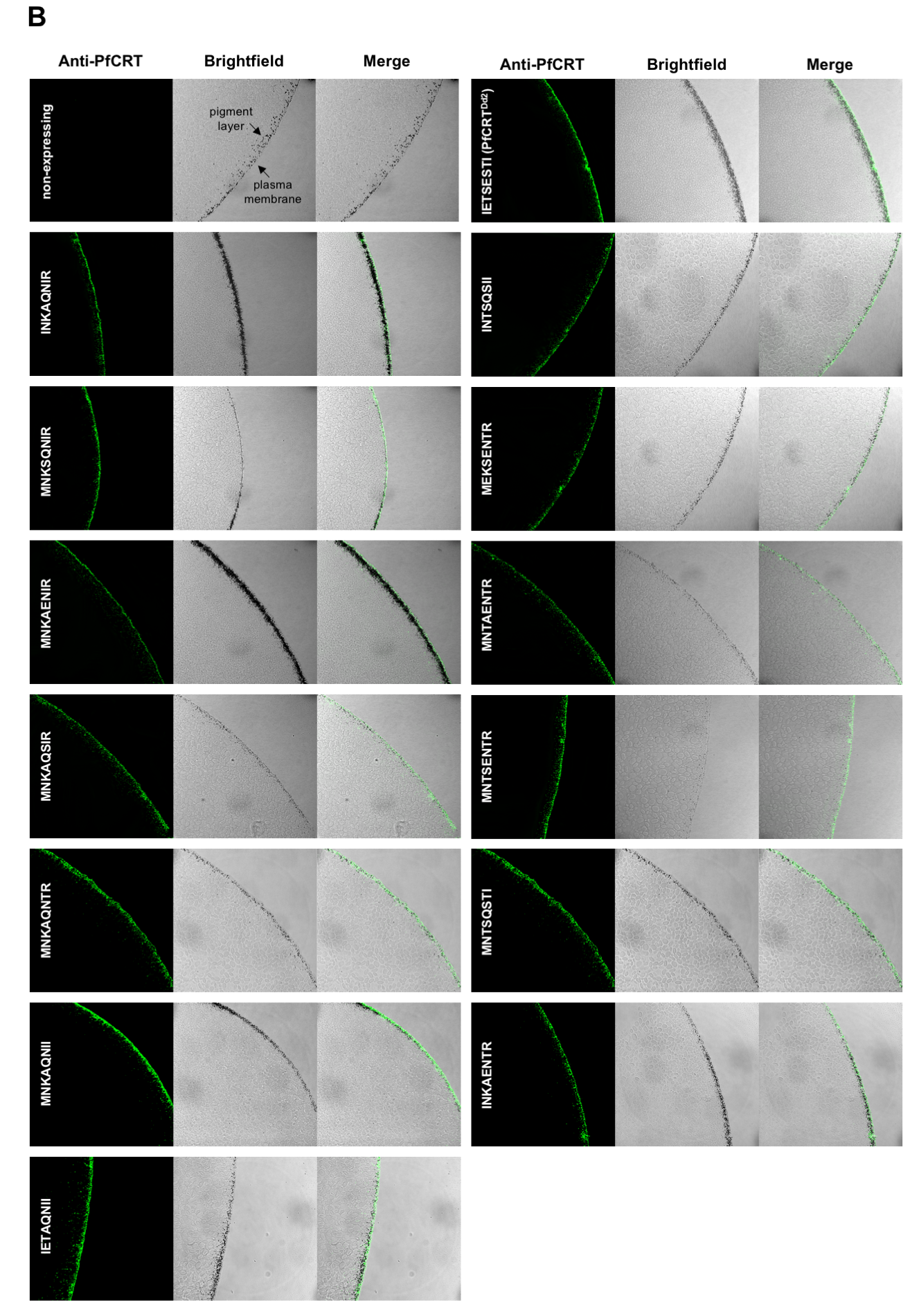

Supplement: S3 Fig — (Similar results for a second frog a shown in S2 Fig). Immunofluorescence microscopy was used to localize those PfCRT variants that exhibited little or no CQ transport activity when expressed in the oocyte system. In each case, the expression of the PfCRT variant resulted in a fluorescent band external to the pigment layer, indicating that the protein was expressed in the oocyte plasma membrane. The band was not present in non-expressing oocytes. Panels A and B show the images from two independent experiments that were performed using oocytes from two different frogs, and within which images were obtained from a minimum of three oocytes per PfCRT variant. Refer to Richards et al. [75] for the localization of IEKSESII (i.e. the ‘106/1’ isoform of PfCRT). (TIF) [file pcbi.1008243.s003.tif]

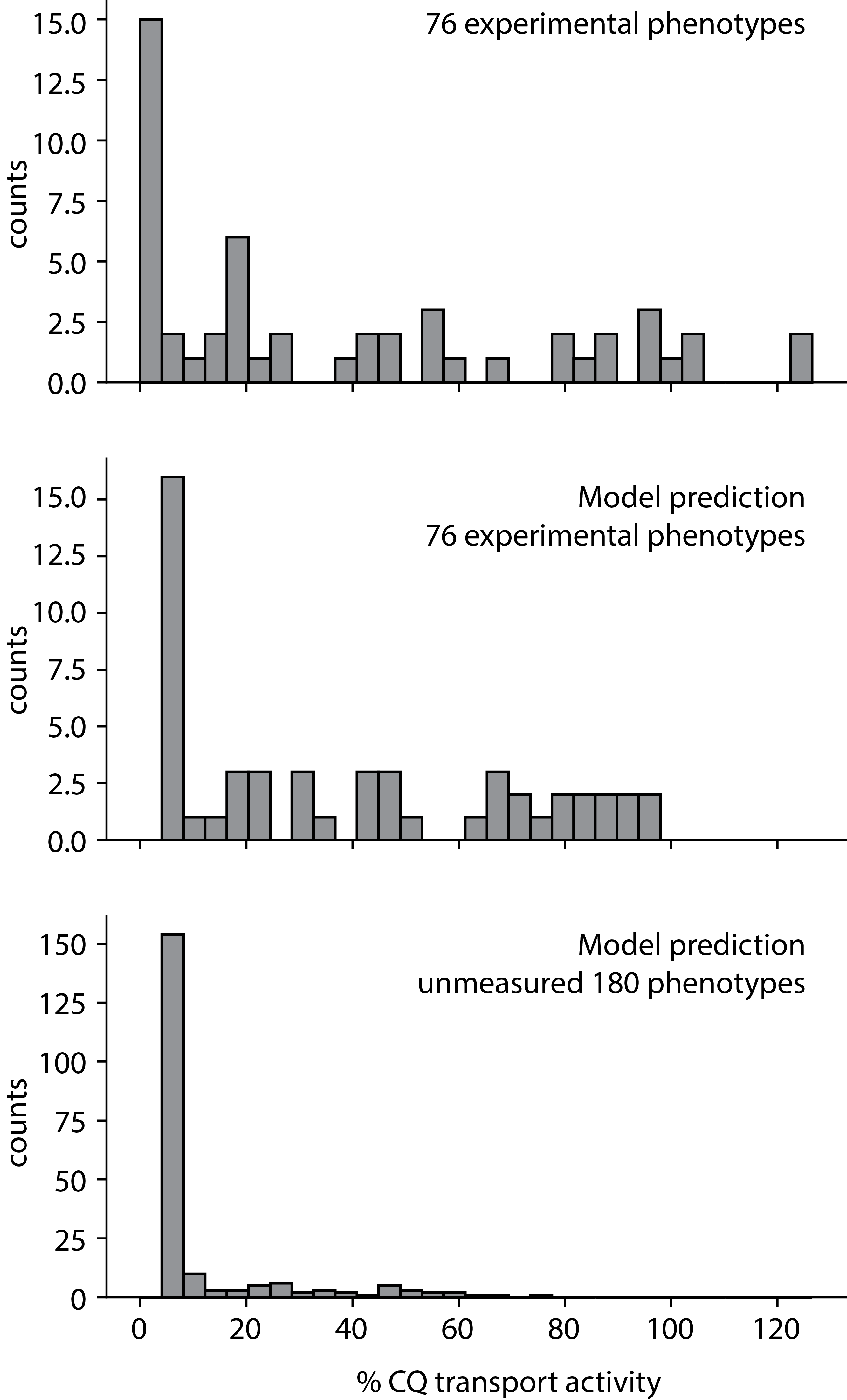

Supplement: S4 Fig — Sub panels show % CQ transport for the 76 genotypes with measured phenotypes (top panel), the model predictions for those 76 measured phenotypes (middle panel) and the 180 genotypes with unmeasured phenotypes (bottom panel). % CQ transport was measured relative to PfCRTDd2. (TIF) [file pcbi.1008243.s004.tif]

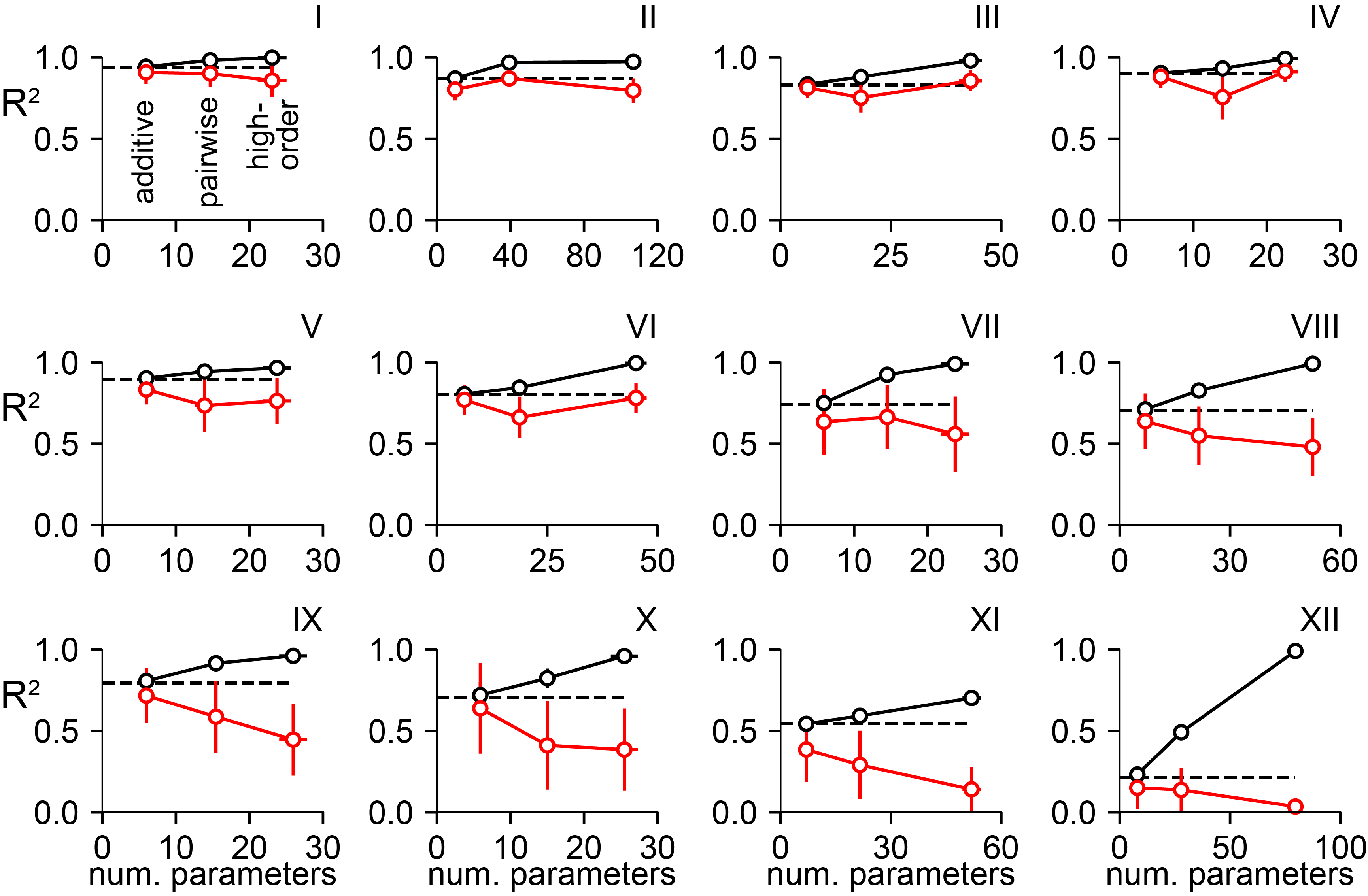

Supplement: S5 Fig — Each sub-panel shows R2train (black) and R2test (red) for the map indicated above the graph (see S2 Table) as epistatic orders are added to the model. The x-axis is the number of parameters used in the fit. Points are, from left to right: additive, pairwise, and high-order epistasis. Points and lines indicate the mean of 1,000 pseudoreplicate samples in which we trained a model on 80% of the genotypes and predicted the remaining 20%. Error bars are standard deviation of pseudoreplicate results. The dashed lines indicate the fraction of the variation in the map explained by the additive model. (TIF) [file pcbi.1008243.s005.tif]
