# Supplementary material for: Inferring a complete genotype-phenotype map from a small number of measured phenotypes
Source: PLoS Comput Biol. 2020 Sep 29;16(9):e1008243. doi: 10.1371/journal.pcbi.1008243 (PMC7546491; doi:10.1371/journal.pcbi.1008243)
Supplement: S1 Table — (DOCX) [file pcbi.1008243.s006.docx]

**S1 Table: Primer sequences used to introduce mutations into the PfCRT coding sequence via site-directed mutagenesis.**

| **Amino acid change** | **Forward primer 5′ to 3′^a^** | **Reverse primer 5′ to 3′^a^** |
| --- | --- | --- |
| I74M | GTGTCatgGAAACAATTTTTGCGAAG | GTTTCcatGACACAAACAGACAGGTAG |
| N75E | GTTTGTGTCATGgaaAAGATTTTTGCG | CGCAAAAATCTTttcCATGACACAAAC |
| E75N | GTCATTaacACAATTTTTGCGAAGAGAAC | CGCAAAAATTGTgttAATGACACAAACAG |
| T76K | gtgtcattaacaagatttttgcgaagag | GCAAAAATcttGTTAATGACACAAACAG |
| S220A | GTATTAATTTCTgcgCTGATCCC | GGATCAGcgcAGAAATTAATAC |
| Q271E | TCTGAAAgaaCTACATCTGCCCTATAATG | GGGCAGATGTAGttcTTTCAGAAAGGG |
| E271Q | CTACCCTTTCTGAAAcaaCTACATCTGCCC | GGGCAGATGTAGttgTTTCAGAAAGGGTAG |
| N326S | CTTCtcgATTTGTGATAATCTGATTACGTC | CAAATcgaGAAGAACGAAAATAACGCGAAC |
| S326N | CTTCaacATTTGTGATAATCTGATTACGTC | CAAATgttGAAGAACGAAAATAACGCGAAC |
| I356T | GCCacaGCGATCGCGTATTACTTC | CGCGATCGCtgtGGCGGGCCCCTG |
| R371I | GGCGATGTTGTGattGAACCCAGACTG | CAGTCTGGGTTCaatCACAACATCG |

^a^Mutated codons are shown in lowercase.
