# Supplementary material for: Inferring a complete genotype-phenotype map from a small number of measured phenotypes
Source: PLoS Comput Biol. 2020 Sep 29;16(9):e1008243. doi: 10.1371/journal.pcbi.1008243 (PMC7546491; doi:10.1371/journal.pcbi.1008243)
Supplement: S2 Table — (DOCX) [file pcbi.1008243.s007.docx]

**S2 Table: Published genotype-phenotype maps used to test method (see S5 Figure).**

| ID | genotype | phenotype | L | reference |
| --- | --- | --- | --- | --- |
| I | genomic mutations | *E. coli* fitness | 5 | [56] |
| II | point mutants | bacterial fitness | 5 | [4] |
| III | chromosomes | *A. niger* fitness | 5 | [14] |
| IV | point mutants | binding affinity | 5 | [10] |
| V | alleles in network | *S. cerevisiae* growth rate | 6 | [57] |
| VI | alleles in network | *S. cerevisiae* growth rate | 6 | [57] |
| VII | genomic mutations | *E. coli* fitness | 5 | [58] |
| VIII | genomic mutations | *E. coli* fitness | 5 | [58] |
| IX | chromosomes | *A. niger* fitness | 5 | [14] |
| X | alleles in network | *S. cerevisiae* sporulation | 6 | [57] |
| XI | alleles in network | *S. cerevisiae* mating | 6 | [57] |
| XII | genomic mutations | *E. coli* fitness | 6 | [28] |
