# Supplementary material for: Inferring a complete genotype-phenotype map from a small number of measured phenotypes
Source: PLoS Comput Biol. 2020 Sep 29;16(9):e1008243. doi: 10.1371/journal.pcbi.1008243 (PMC7546491; doi:10.1371/journal.pcbi.1008243)
Supplement: Attachment — Submitted filename: cover-letter.pdf [file pcbi.1008243.s011.pdf]

UNIVERSITY OF OREGON

April 24, 2020

Dear Editor:

Thank you for considering our revised manuscript for publication in *PLOS Computational Biology* and for obtaining such thoughtful reviews. Addressing their concerns has improved both the manuscript and the software. In this revision, we have:

- Added analyses of simulated genotype-phenotype maps to better understand the nature of the inference and resulting uncertainty in our predictions.
- Added analyses of 13 published genotype-phenotype maps to establish the generality of our approach, as well as its scalability to large genotype-phenotype maps.
- Improved our software to allow end-users to better understand the inference that results when applied to their own datasets.
- Updated the software documentation to improve usability and transparency for the end-user.
- Edited the text to add these new sections and address issues raised by the reviewers.

These changes are documented in a point-by-point response to the reviewers below. We hope that this set of changes adequately addresses the concerns raised by the reviewers and that the manuscript is now acceptable for publication in *PLOS Computational Biology*.

Sincerely,

Michael J. Harms, PhD
Associate Professor of Chemistry and Biochemistry
Institute of Molecular Biology
https://evolutionarybiochemist.org
harms@uoregon.edu
541-346-9002

An equal-opportunity, affirmative-action institution committed to cultural diversity and compliance with the Americans with Disabilities Act

We have copied the reviewer comments (slightly reformatted and numbered) below. Our responses to the comments are in blue. For convenience, we have also copied in some direct quotes from our revised manuscript. These are shown in orange. We have also included a pdf with all changes tracked and annotated as an "Other" file with the resubmission.

Reviewer #1:

I find this article promising, but I have some concerns, mainly regarding the software, which need to be addressed before I can recommend it for publication. Since the primary subject of the article is a model for which the authors have provided code as implementation, it is reasonable that the software should be subject to the same scrutiny as the written article.

Thanks for the feedback on the software from the perspective of a potential user: it's extremely helpful. We address the reviewer's specific concerns below, but as an overall response, we have made three major changes. First, we have updated the software documentation extensively, both through the addition of a more helpful `--help` call on the command line and through a massive update to the documentation (https://gpseer.readthedocs.io). This includes a full tutorial (https://gpseer.readthedocs.io/tutorial.html). Secondly, we have updated the command line and user inputs to be simpler and—we hope—more intuitive for the user. Thirdly, we have modified the software to automatically return files and plots to help the user decide whether the model fits the training data well, if adding more data would be helpful, and whether the model is predictive (at least, in a cross-validation context).

Serious concerns:

1. The software requires the user to input a csv file with several required fields, not all of which may be used in any particular run. I think that the help documentation should make it clear that for the run to be successful, the input csv file must include fields such as `nreplicates', `stdeviation', etc.

Done. Please check out our new `--help` string and the new `Input file` section of the documentation (https://gpseer.readthedocs.io/en/latest/io.html). In fact, we removed these column requirements; now, the software only requires the genotypes and phenotypes (as documented).

2. After reading the software documentation, and skimming through the code, I was disappointed when I realized that there was no obvious procedure for testing input where fitness (or fitness proxy) values were known but could be hidden from the algorithm with the purpose of doing a "goodness-of-fit" test. Specifically, I would have liked to submit, along with the input, a csv file containing the test genotypes along with their true but hidden-from-the-algorithm fitness values. The procedure should then output a copy of these two columns along with a column showing the predicted fitness values. Any other summary statistics related to fit and deemed worthy could be included in this output as well.

This is a great suggestion. In fact, the software already did part of this procedure, but in a poorly documented fashion. We have modified the software so it does three tasks differently from the previous version:

1. It returns both the predicted values for the inputs and the predicted values for the outputs. This allows the user to easily determine if the software is fitting the existing data well.
2. We added a cross-validation mode in which it predicts on subsets of the input data. This allows the user to estimate how well the model is performing in predicting unmeasured data.
3. We have made the process much easier and more transparent for the user. The software now automatically generates a selection of graphs to help the user assess how well the analysis is

performing. We also added a tutorial (https://gpseer.readthedocs.io/tutorial.html) that walks the user through how to undertake an analysis both using the command line and API.

3. I'm aware that the experienced user could easily dig through the backend packages (also produced by the authors) or copy and paste with various csv files and obtain what I describe above. However, given that the main point of the article is to tout the _predictive_ power of the model, I think it is important that the user have an immediate "front-end" feature for submitting data with known but hidden fitness values, and getting an analysis of how well the model did in guessing these values.

We agree and hope the changes documented above now achieve this aim.

Minor concerns:

4. The software provides an option for submitting a "--genotypes_file". I think that the help documentation should be more clear that this is not expected to be a csv file, but rather a plain text file with the list of genotypes for which values are to be predicted. This of course ties in with my concern expressed above.

Thanks for the suggestion. We have updated the --help documentation.

5. CQ transport activity plays the role of a fitness proxy, but it is remarked that the derived 8-mutant is not the most "fit", i.e. there are reductions in transport activity in some of the implied paths from wildtype to 8-mutant. Since this is the case, why are only forward paths considered? It seems plausible that some of the random landscapes generated in the simulations may contain evolutionary paths which are not forward paths. I realize that this implies analyzing a far larger set than 8! = 40320, but perhaps a random sampling of paths could be used for this purpose rather than exhaustively covering all possibilities.

This is an interesting suggestion, but, given the complexity of the manuscript and the analysis as written, we have decided not to pursue non-forward trajectories in this manuscript. We are careful in our language to indicate that we are discussing forward paths only.

Nitpicks:

6. When reading the paper, I found it irritating to have to flip back and forth between pages to locate where a genotype is located on the fitness landscape. Throughout the text, I recommend attaching bitstrings to each phenotype so the reader can see immediately where it lies, without having to refer to the page containing the fitness landscape diagram. For example, g84 could be written as "g84 (11111101)" since it is third to last in the row corresponding to 7-mutants in Figure 6.

This is a great idea. We have implemented this throughout the text and figures.

Subtler concerns:

7. This is a "science/philosophy" point, but I'm leary about the mixing of non-parametric methods with parametric methods. Any curve can be "straightened out" via non-parametric methods like splines or loess curves. But what structures might be getting wiped away by this smoothing? It's true that if we are judging a model solely by its predictive power, then naturally the final test is how well it predicts the missing data. But surely by "straightening out" curvature via splines we risk missing structure which could lead to a deeper understanding of the mechanisms underlying the phenomenon we are exploring. (Pre-Newtonian physics come to mind, I sometimes feel that we are in a similar state in evolutionary prediction.)

Nevertheless, the authors' point that after smoothing epistatic coefficients lend no additional predictive power, and even worsen it, is an important observation, particularly given the popularity of the concept of epistasis in this field.

In the section on improvements, the authors make a brief comment which addresses the point that I am trying to make here. So this item is more of a general reflection rather than anything that needs to be fixed.

We agree with the reviewer about the unsatisfying notion of smoothing out the data with a non-parametric method. (The Harms lab is developing mechanistic, predictive models to account for such nonlinearity—this work will be forthcoming.) That said, when it comes to prediction, the combination of a non-parameteric and parameteric model seems the most effective. We draw attention to the following line in the discussion: “This is a powerful approach to identify the curvature in data, but the spline is an empirical parameter that has no intrinsic, mechanistic meaning. Ultimately, it would be more desirable to define a model that describes the mechanism underlying the nonlinearity in the map.”

Final note

If the concerns above are addressed, I think the resulting paper and accompanying software would be a fine contribution worthy of publication.

Thanks!

Reviewer #2:

Sailer et al. develop and analyze a method called GPSEER. This method incorporates the additive effects of mutations, a binary classifier, and a nonlinear scale, and is designed to predict missing phenotypes from an incomplete combinatorial genotype-phenotype map. One of the most impressive features of the method is its ability to characterize the uncertainty of its predictions, which facilitates the characterization of uncertainty in evolutionary forecasts. Sailer et al. apply their method to a previously published combinatorial genotype-phenotype map for the phenotype of chloroquine (CQ) transport activity, which is important for the evolution of drug resistance in the malaria parasite. They validate their model by using it to predict the CQ transport activities of 24 new genotypes, which were measured for this study. They then study the complete, predicted map to make evolutionary inferences about the evolution of chloroquine (CQ) transport activity in nature. The paper is exceptionally well written, clear, interesting, and of direct relevance to the readership of PLoS Computational Biology. The methods are also made freely available as a command line tool and API. I commend the authors for their work.

My main concern with this paper is that it is about a new method, but this new method is only tested on a single genotype-phenotype map. I understand that this particular map is an important one to study and that in applying their method to this map, the authors have gained important insight into the evolution of chloroquine (CQ) transport activity. This is all good. But if this paper is about a new method, what have we learned about this method's predictive power outside of this particular genotype-phenotype map? How does it perform on other genotype-phenotype maps? Does its performance depend upon topographical properties of the map, such as its overall ruggedness? The authors state that their method "should be applicable to a large number of genotype-phenotype maps", but whether or not it is remains to be seen.

I have detailed this major concern below, as well as some other minor concerns. Having been on the other side of a review like this before, I completely understand if the authors disagree with and are frustrated by my assessment. I therefore leave it up to them to decide if the incorporation of more genotype-phenotype

maps is necessary to fairly assess the performance of their method, but if they decide it is not, then I kindly ask for a justification.

Thanks for the thoughtful review.

Major:

1. My one major criticism of this otherwise excellent work is that the authors only study one combinatorially complete genotype-phenotype map, when many others are publicly available. For example, Bank et al. (2016) "On the (un)predictability of a large intragenic fitness landscape" PNAS. In some cases, complete landscapes are available, which could be subsampled to create combinatorially-complete landscapes in replicate. For example, Wu et al. (2016) "Adaptation in protein fitness landscapes is facilitated by indirect paths" eLife. Alternatively, or additionally, the authors could apply their method to simulated combinatorially complete genotype-phenotype maps. By applying their method to these or other combinatorially-complete landscapes (those listed above are just a few of many examples) the authors could better assess its performance.

Thanks for bringing this up. We have addressed this critique in three ways in the revised manuscript.

First, we added a section ("Model convergence and uncertainty") where we simulated sampling combinatorially complete genotype-phenotype maps and then tested our ability to infer unmeasured phenotypes with increasing numbers of observations (new Figure 5). This allows us to better articulate the nature of the inference, why the model converges with the number of observations made, and better understand the nature of the uncertainty in the predictions.

Second, we added a section ("Generality of the model") where we tested the behavior of the approach on 13 published genotype-phenotype maps. Twelve of the maps are of similar scale to the PfCRT map and serve to demonstrate that the finding that epistatic coefficients lead to poor predictive power is a common feature of such maps. This result gives us confidence in our selection of model. The final map is a large, combinatorial map of dCas9 binding targets. We test the predictive power of the model and find we can fully train the model with ~4,000 observations—allowing us to predict the remaining 55,000 observations with known uncertainty. This gives us confidence that the analysis approach scales well to larger genotype-phenotype maps.

Minor:

2. An important reference is missing: du Plessis, Leventhal, & Bonhoeffer (2016) "How good are statistical models at approximating complex fitness landscapes" Mol Biol Evol 33, 2454-2468. I think this could be discussed in the final paragraph before the discussion section "Future improvements". Also, depending upon the authors' and PLoS' policies regarding the citation of pre-prints, this highly relevant paper could be discussed: Zhou & McCandlish (2019) "Minimum epistasis interpolation for sequence-function relationships" bioRxiv.

Thanks for bringing this to our attention. We have added the following paragraphs to the discussion:

Under the "Global features, not local epistasis, are useful for prediction section":

A powerful aspect of using a global model, such as a spline, is that it normalizes the fit residuals. This approach gives all phenotype predictions the same, normally distributed uncertainty (Figure 5A). These distributions can then be sampled to understand, in a statistical sense, what features of the map shape evolutionary trajectories independent of prediction uncertainty. This method sidesteps a key issue in

completing partially measured genotype-phenotype maps: we will *never* have sufficient information to predict unmeasured phenotypes with perfect accuracy, regardless of sampling strategy [64].

And under the "Future improvements" section:

Finally, a recent paper has identified a regression strategy that yielded predictive epistatic coefficients [72]. Such strategies, in combination with approaches such as classifiers and nonlinear splines, could allow the extraction of further information—and thus the development of better predictive models—from large genotype-phenotype maps.

Trivia:

3. (btw, it is very annoying to not have page numbers or line numbers in this manuscript)

Apologies for that oversight. An earlier draft of the manuscript had both line and page numbers…which were somehow lost. We agree that would have been annoying.

4. In the caption of Fig. 2, the yellow line for the spline fit in B is not described in the caption.

We have now updated the caption to read. The yellow line in panel B shows the spline fit to the data.

5. In the main text, the authors mix up the red arrows and blue arrows when describing Fig. 2

Thanks, fixed.

6. In the references, some titles have all words with their first letter capitalized, others just the first word. Please fix.

Thanks, fixed.

Reviewer #3:

In their manuscript entitled "Inferring a complete genotype-phenotype map from a small number of measured phenotypes" the authors present an approach to predict a complete genotype-phenotype map from an observed subset. The genotype space consists of all 256 mutational combinations of eight particular amino-acid changes of the malaria parasite's PfCRT transporter that distinguish the wild-type 3D7 variant from a Dd2 isoform with increased chloroquine (CQ) transport activity, which in turn leads to greater CQ resistance of the parasite.

This is interesting and timely work, and the manuscript is well-written and mostly clear.

The CQ transport phenotype has been measured for 52 of the 256 genotypes in previous work. The authors use these results to train predictive models of increasing complexity until they reach an R^2 of 0.97 with a 70-parameter model. There is always a danger of overfitting with so many parameters (particularly when the number of observations is relatively small) and the authors duly report that an additional 24 observations were not predicted well by this complex model. A rather simpler model performed better, which means that the large R^2 for the 70-parameter model was indeed due to overfitting (as the authors also conclude).

The authors then try to show that the choice of these particular 52+24 observations does not bias the predictions by constructing random samples of 52 from these 76 and recalculating their model.

This is not entirely convincing, since the 24 were not randomly chosen from the remaining 204, but were strongly influenced by the model predictions based on the 52. It would have perhaps been advisable to select an additional random sample. Moreover the random samples that are performed show two slightly worrying features, namely that the nonlinear part of the model no longer improves anything for the random samples (indeed decreases the performance very slightly), and also that the R^2 now is somewhere around 0.70 (red curve in Fig. 4B). The fact that the nonlinear part does not add much is also not too surprising if one compares Fig. 2G and 2H, which look almost identical despite going from R^2 = 0.78 to R^2 = 0.90. The main difference between the two is the outlier around position (-30,5), which may well affect the R^2 quite strongly. But the sensitivity of R^2 and indeed the model training process to such an outlier decreases the confidence one can have in this approach.

The predictions for the 180 remaining genotypes appear to largely be quite low. This may of course be accurate, but given the problems with the model outlined above, and also given that the model may systematically underestimate large phenotype values (Fig. 2G & 2H) I worry that most of the 180 are simply predicted to have low phenotype values because they are far away from the training set. Most of the regions of the genotype map with high transport activity in Fig. 5 can already be anticipated in Fig. 1.

Thanks for the careful read of our data and for the thoughtful critique. As we interpret this, there are two key main issues raised: 1) the *training set* of 52 data points was non-random and could have biased the results and 2) the *test set* of 24 points was also non-random and could have given a false picture of model predictive power.

1. We agree that, in principal, fully random sampling for the initial training set would have been optimal from a pure method-development standpoint. Practically, however, we must work with the starting point from the previous work--a nonrandom sampling of the map (see below for how we have altered our discussion of the preexisting data). We have clarified how these genotypes were selected:

   These genotypes were selected with the specific goal of identifying possible trajectories between $PfCRT^{3D7}$ and $PfCRT^{Dd2}$ [24]. These genotypes were scattered through the map, with two single-, seven double-, nine triple-, ten quadruple-, seven quintuple-, four sextuple-, and eight septuple-mutants.

   To overcome this practical difficulty, we have expanded the model validation work extensively (see our response to reviewer 2, point #1). As such, the PfCRT data are only one part of the overall validation of the approach. The new validation includes simulations (to better flesh out the meaning of convergence and prediction uncertainty in the analysis), analyses of new combinatorially complete genotype-phenotype maps, and a new analysis of a large, sparsely sampled map. All of the analyses point to the same basic conclusion: a nonlinear spline with an additive model is the most predictive and has well-defined error.

2. We have better described the nature of the test set and our rationale for including the genotypes we chose. The test set was not entirely random, but if anything it is biased towards genotypes that are distant from the known data, rather than the known data:

To test the predictive power of the model, we selected 24 uncharacterized PfCRT genotypes and measured their CQ transport activities using the *Xenopus* oocyte system (Figure 3). To create this set, we randomly selected 18 genotypes from the center of the map that contained between three and six

mutations. We completed the set of 24 by adding the six previously unmeasured single-mutation genotypes. We included these genotypes because the model predicted that none of these genotypes would possess CQ transport activity. We wished to test this prediction because it implies that the first evolutionary step from $PfCRT^{3D7}$ did not improve CQ transport activity. The 24 new test genotypes, which are outlined in yellow in Figure 5A, consisted of six single-, three triple-, five quadruple-, five quintuple-, and five sextuple-mutants. Eleven of the genotypes were predicted to lack CQ transport activity; the remaining 13 were expected to possess between 9% and 74% of the activity exhibited by $PfCRT^{Dd2}$.

Information that (as far as I can see) is missing, is:

- where are the 24 observed phenotypes in Fig. 5? In general I find it somewhat misleading that the predicted and observed values are not distinguished. We should clearly be able to see the 52+24+108.

Great suggestion. We have updated the figure accordingly. The genotypes corresponding to the original measurements are now indicated with black outlines; the new measurements are outlined in yellow; the predictions are the remainder.

- what governs proximity in the representations used in Figs. 1 and 5? Is it in any way related to sequence distance (presumably, looking at the lines connecting the nodes, which seem to be minimised in length?) If so then this contributes to my worry, since we simply see the red regions in Fig. 1 grow a bit, which means that only genotypes close to observed phenotypes with high phenotype values are predicted to have high phenotype values as well.

There is no simple map between sequence distance and position on each of these graphs. Because this is a low-dimensional projection, many of the edges between rows connect visually distant genotypes. We have clarified this in the text, adding the following to the Figure 1 legend:

Genotypes are in sorted combinatorial order, left to right, such that the second row contains 1000000, 0100000, ... , 00000010, 00000001, the third row contains 11000000, 10100000, … , 00000101, 00000011, the fourth row contains 11100000, 11010000, …, 00001011, 00000111, etc.

- what is the distribution of predicted values for the 180 genotypes versus the distribution of the 52+24?

We have added this as a supplemental figure (S4 Fig). We found that the training data had a slightly larger fraction of active genotypes relative to the final predictions for the whole dataset.

In conclusion my worry is that the model is overly reliant on the 52 original observations (the selection criteria of which are also not discussed much). Based on the fact that the additive model with classifier is basically the best model here, and that the 24 additional observations are chosen with that rationale of selecting "genotypes with different numbers of mutations that were predicted to exhibit substantially different levels of CQ transport activity" an additive model will likely only find substantially different levels of transport activity in the proximity of genotypes already exhibiting that activity. The 24 are therefore highly dependent on the 52, and the additive nature of the model means that we can't have high confidence that there isn't another pocket of high transport activity hiding among the 180. I think there's a good chance there indeed isn't, given that a third of genotype space has already been observed, but the authors clearly envisage applying this method to much larger spaces, where such hidden pockets would be much more likely.

Hopefully, in the work above, we have addressed these concerns adequately. Our simulations show when we would expect to see convergence of the additive model, which matches what we observed from our (slightly) non-random sampling. We clarified the nature of the sampling of both our experimentally measured training and test datasets. Finally, we validated the model with 13 other genotype-phenotype maps.

It is interesting that the additive model is so successful - I do believe this is probably a useful model - and a pity in a way that the authors start with such a large set of observations and add further observations that aren't truly independent. One could imagine an approach that starts with a random sample of observations that covers the genotype space in a fairly even way and then trains a model using this data and tests it on a further random sample before filling in the space completely.

One way to further validate their method in this particular case would be to measure the phenotype for a random sample (uniform in genotype space) of the 180, but I realise that this is probably a lot of additional work.

As noted above, we did this computationally, demonstrating that convergence is determined by the number of genetic backgrounds in which each mutation is observed and the magnitude of the epistasis in the map.

If the authors can provide convincing arguments that allay the above concerns, I'd be happy to recommend this article for publication.
