# Supplementary material for: Inferring a complete genotype-phenotype map from a small number of measured phenotypes
Source: PLoS Comput Biol. 2020 Sep 29;16(9):e1008243. doi: 10.1371/journal.pcbi.1008243 (PMC7546491; doi:10.1371/journal.pcbi.1008243)
Supplement: Attachment — Submitted filename: cover-letter.pdf [file pcbi.1008243.s012.pdf]

UNIVERSITY OF OREGON

July 10, 2020

Dear Editor:

Thank you for considering our revised manuscript for publication in *PLOS Computational Biology*. We have made the textual changes requested by the reviewers. These changes are documented in a point-by-point response to the reviewers below. We hope that this set of changes adequately addresses the concerns raised by the reviewers and that the manuscript is now acceptable for publication in *PLOS Computational Biology*.

Sincerely,

Michael J. Harms, PhD
Associate Professor of Chemistry and Biochemistry
Institute of Molecular Biology
https://evolutionarybiochemist.org
harms@uoregon.edu
541-346-9002

An equal-opportunity, affirmative-action institution committed to cultural diversity and compliance with the Americans with Disabilities Act

We have copied the reviewer comments (slightly reformatted and numbered) below. Our responses to the comments are in blue. For convenience, we have also copied in some direct quotes from our revised manuscript. These are shown in orange. We have also included a pdf with all changes tracked and annotated as an "Other" file with the resubmission.

Reviewer #3:

3.1: It is unclear to what extent the phenotypes in the 12 maps mentioned in Table S2 were measured. Were they all mapped fully? If not it would be nice to see the extent in the table.

Yes, they were fully mapped. We clarified this in the text (bold below):

We therefore tested the models we investigated for the PfCRT dataset on 12 previously published, **exhaustively measured**, binary genotype-phenotype maps

3.2: Are the parameters for the application of the model to these other GP maps given somewhere?

We have added a new file (S3 file) that has the measured and predicted values for each of these genotype-phenotype maps and have referenced it in the text. We also modified S5 Figure and its legend to make the our analysis more clear (with the new text shown here in bold):

We then fit progressively more complex models to each dataset, as we did in Fig 4 for the PfCRT model. We observed similar behavior to all of these datasets: a nonlinear model with an additive scale gave the highest predictive power. Addition of epistatic coefficients led to reduced predictive power in every map we tested (S5 Figure). **The fit results are reported in S3 File**. This suggests that our approach—using a nonlinear model coupled to additive mutational effects—can be applied to other genotype-phenotype maps.

3.3: The references in line 4, p.19, "[4,10,28,57–60]" aren't the same set of references as mentioned in Table S2, which are [4,10,19,59,60,83,84]. Is this correct?

Good catch! We had a mix up in our reference library software. This issue has been fixed.

3.4: For the 13th, much larger map, how were the training and test sets chosen with respect to the underlying (skewed) distribution of the 59,394 oligonucleotides? Would different samplings that either (a) mirror the skew or (b) try to mitigate it by sampling in a way that ensures equal representation of the bases where possible change the results, and what would that mean for the method?

The training set was sampled randomly from the observed genotypes, hence it mimics the skew in the data. We did consider alternate sampling methods to try to remove the skew, but decided that such an analysis was outside the scope of the current investigation. We predict that skew will not have an effect, but it would take a significant amount of work to demonstrate this convincingly. As it is, we make no claim one way or the other about the effect of skew—which we think is appropriate given the evidence we present. To clarify the state of our knowledge in this regard, we added the following two sentences to the relevant section:

This analysis was done in the context of training and test sets that were both sampled randomly from the same set of biased set of measured genotypes (Fig 8A). The predictive power of the model for a completely random, unbiased sample of genotypes—and the general effect of bias in the training set on model power—remains an open question.

The reason we expect a small effect from skew is that: 1) We linearized the data and then 2) We fit an additive model to the linearized data. One way to think of the linearization is as converting epistasis into a random normal variable applied to the additive phenotype of each genotype. When we fit an additive model to the data, all that will matter is our ability to resolve the additive effect of each mutation over the noise induced by the epistasis. A skewed sample would mean we need to sample more genotypes to see every mutation enough times to resolve its additive effect, but it would not introduce bias into the predicted phenotypes.

If skew does have an effect, we would predict bias in our predicted phenotypes. We see no evidence for this, suggesting skew is not having an effect. There could, however, be subtle issues with regard to distortion in the nonlinear spline, or less-subtle issues that arise because we sampled a non-random sub volume of the total map. To probe this adequately, we would need to do simulated sampling with different amounts of skew, artificially sample the genotypes from the existing map to remove skew, and do a wide variety of statistical tests to explore output bias. This is a large (important) can of worms, but is more than we can adequately address in the current manuscript.

Reviewer #4:

4. 1: (Important) It is not clear to me how the probabilities for trajectories were computed (for instance how suboptimal peaks were handled). In particular, the sentence “We used a selection coefficient of +0.1 for . . . “ is unclear to me. I would appreciate if the authors could make it crystal clear how probabilities were computed in the text (I don’t think readers should have to consult the software package or other papers.)

We have updated the methods accordingly to clarify how we converted %CQ transport to relative fitness. We expanded this line:

We used a selection coefficient of +0.1 for $PfCRT^{Dd2}$ [100% CQ transport activity relative to $PfCRT^{3D7}$ (0% CQ transport activity)].

Into this:

We assigned fitness values to each genotype by assuming a linear relationship between CQ transport activity and selection coefficient. We used the formula: s = 0.1 x % CQ transport activity. We used the $PfCRT^{3D7}$ genotype as our reference to calculate relative fitness. Thus, relative fitness of $PfCRT^{3D7}$ (0% CQ transport activity) was 1.0, while the relative fitness of $PfCRT^{Dd2}$ (100% CQ transport activity) was 1.1.

4.2: The authors do not consider reversed mutations. I’m fine with that, but I think that at least a brief comment would help. Why is the simplification [not to consider reversed mutation] reasonable? How can we have some confidence in the probabilities anyway? I have noticed that this rather daring simplification is far from rare in the literature, usually without justification.

This is a good point. As we noted in the initial text, our calculated probabilities are a sort of “null model” that allows us to ask whether a simple evolutionary scenario can reproduce the genotypes observed in the field. This simple scenario is: selection only for improved CQ transport alone, forward steps only, and strong-selection/weak mutation. We conclude that such a model is not sufficient to reproduce the environmental isolates of PfCRT—other factors must be in play. We have clarified that the limitation to forward steps is an assumption of the model by adding the bold sections to the text below.

These trajectories can be thought of as a null model, as the analysis does not account for other components of fitness, such as the requirement for the protein to maintain its natural function as it evolved into a bi-functional transporter, **nor does it allow reversions along the evolutionary trajectory.** A comparison of these predicted pathways with the fragments of PfCRT's mutational routes that can be inferred from the combinatorial genotypes – e.g., the PfCRT$^{China\ e}$ [11111000] and PfCRT^{GB4} [11111001] isoforms – detected in field isolates will aid in identifying where mutations are likely to have become fixed for reasons other than the **stepwise** optimization of CQ transport.

4.3: I greatly appreciate the discussion about a general framework for classifiers under "Future Improvements". However, I kind of wondered about a general procedure for classifiers throughout my reading of the manuscript! Please either comment on a general framework earlier, or point out that the topic will be discussed under "Future Improvements".

Good suggestion. We have added a sentence where we introduce the logistic classifier in the results.

(Although we used a logistic classifier here, there are a number of ways such a classifier could be constructed; see Discussion).

4.4: (Optional) Several methods that use some version of "global epistasis" have been proposed in recent years. It would be great if the authors could provide a little bit of context, such as a brief discussions about how the proposed method compares to published methods (including the role and importance of the binary classifier).

Thanks for the suggestion. We added two sentences to briefly discuss how the classifier related to the nonlinear function:

This is consistent with previous observations about global features of genotype-phenotype maps [45,46]. Previous approaches to this problem have used a nonlinear function to describe global features [45,46]; by adding a classifier on top of the nonlinear function, we better described the underlying distribution of function in the map and therefore gained predictive power.

Minor:

4.5: Author summary, Line 15: "gain robust insight" sounds a bit strange.

Changed to "gain insight"

4.6: Introduction, Line 3: "accessibility of adaptive evolutionary trajectories" (wording)

Thanks for suggestion, but left in place.

4.7: Introduction, Line 5: Did you intend to cite [21,22] here?

No. This was our intended pair of citations, as 20-21 focus on changes in population structure, while 22 is specifically about enzyme genotype-phenotype map.

4.8: After "Testing the model with newly-measured phenotypes", Line 21: "Figure 5A" should be 6A.

Thank you, this has been fixed.

4.9: Above “PfCRT prediction and uncertainty”, Line 6: Check symbol.

Thanks you, this is correct.

4.10: Page 36, Line 14: The summation subscript for the third summation sign needs a correction (should be $k < j < i$).

Thank you, this has been fixed.
